# Analysis of a Case of Imperfect Bisexual Hermaphrodism in Man

**Published:** 1870-03

**Authors:** E. Goujon

**Affiliations:** Laureate of the Faculty of Paris. From Robin’s Journal de l’Anatom. et de la Pysiol., etc.; Paris


### Original Translations.

ARTICLE I.—*Analysis of a Case of Imperfect Bisexual Hermaphrodism in Man.* By DOCTOR E. GOUJON, Laureate of the Faculty of Paris. From Robin's Journal de l'Anatom. et de la Pysiol., etc., Nov. and Dec., 1869.

IN the course of the month of February, 1868, a young man employed in the administrative department of a railroad, committed suicide by carbonic asphyxia, in a mean apartment upon the fifth floor of a house in the Rue de l'Ecole de Médecine. M. Regnier, one of the municipal physicians, and the commisary of police of the district, informed of the fact, repaired to the residence of the suicide, and found upon a table a letter left by him, in which he stated that he had sought death to escape the sufferings which constantly beset him. These gentlemen, from the external appearance of the body, and from the information which they gathered from the janitor of the building, who saw the young man every day, suspecting nothing which could indicate the sufferings to which he alluded, concluded to examine the genital organs, supposing that he might be suffering from some syphilitic affection which, as is well known, frequently plunges its victims into profound marasmus and great mental depression, which often drives to suicide certain subjects already naturally melancholic.

M. Regnier, at this examination, detected immediately a remarkable anomaly of the external genital organs, and recognized a case of masculine hermaphrodism of the most striking character. In truth, it would be difficult, as will be seen by what follows, to find a mixture of the two sexes carried to a greater extent, in all that pertains to the external genital organs. I was informed of the case by Dr. Duplomb, who, like myself, regretted that the incident should be lost to science, and together we urged M. Regnier to use all his influence with the commissary of police, to induce him to permit us to make an autopsy, and to remove the different parts involved in the anomaly. This permission was granted to me on condition that there should be associated with me some physician having official position ; the presence of M. Houel, adjunct to the Faculty, was secured, to whom, as well as to Dr. Regnier, my thanks are due for the disinterestedness with which they abandoned to me completely the investigation of this remarkable case. The case which I shall report is certainly one of the most complete of its kind which science possesses. Since the individual, who was its subject, can be traced up so to speak, from his birth, until his death, and since the examination of the body as well as the autopsy could be made with all the care desirable. The report is especially complete, from this exceptional fact, that the subject took care to leave us long memoirs, in which he initiates us into all the details of his life, and of all the impressions which were produced in him, at different periods of his physical and intellectual development. These memoirs * have so much the more value, inasmuch as they emanate from an individual possessed of a certain amount of culture (he had a certificate as instructress, and had received the first place in the competition at the Normal school for its diploma) and endeavoring to account for the different impressions which he experienced. The position of this person is not without its parallel. Indeed, there may be found in Geoffrey de St. Hilaire, observations which exhibit great analogy with those which I report.

The hermaphrodite, whom we are discussing, was inscribed upon the civic registers as belonging to the female sex ; he was

* M. Tardieu having become possessed of these memoirs, has, with his usual complaisance, consented to communicate them to me.

reared with young girls, in the midst of whom he passed his infancy and his youth. The physical modifications which were developed subsequently forced him to demand a correction of the civic registration, which definitely returned him to his own sex, which was masculine, although upon a superficial examination of the external genital organs one would have been much more disposed to class him amongst the women: we quote a passage from his memoirs, in which he enumerates rapidly his different positions: "From my arrival in Paris dates a new phase of my double and perplexing existence. Reared during twenty years in the midst of young girls, I was at first, and during two years at most, chambermaid; at sixteen and a half I entered upon the situation of assistant teacher (*éleve maitresse*) at the Normal school of ——; at nineteen years I obtained my certificate as instructress (teacher's certificate); some months after I directed a boarding-school of some note in ——. I left it at twenty-one years; this was in the month of April; at the end of the same year I was in Paris on the —— railroad."

The autopsy which we were enabled to make, enabled us to correct the first decision made as to his sex during the greater portion of his life, and to confirm the accuracy of the diagnosis, which had, in the last place, replaced him in his true social position.

After what has been said, it will be seen that the present case raises many physiological and medico-legal questions. The conformation of the external genital organs of this individual permitted him, although he was manifestly a man, to play in coition the part of a man or woman indifferently; but he was in either case sterile. He was enabled to perform the part of a man in this act, by means of an imperforate penis susceptible of erection, and attaining at that time the dimensions of the penis of many regularly conformed individuals. As will be seen later, by its description, this organ was rather a voluminous clitoris than a penis; indeed, sometimes the clitoris in woman will attain the size of the index finger. Erection could be accompanied by ejaculations and by voluptuous sensations, as he informs us in his memoirs. This ejaculation did not occur from the penis which was imperforate, as has been already indicated. A vagina, terminating in a *cul-de-sac*, and in which the index finger might be inserted without resistance, permitted him to play equally well the part of a woman in the act of coition. To this vagina, situated as it is ordinarily in woman, were annexed vulvo-vaginal glands opening on each side of the vulva, and by the side of the opening of two other small conduits, serving for the emission or ejaculation of semen.

I had made the anatomical description of the subject of this report, when I learned from M. Professor Tardieu that this unfortunate had been the subject of a medico-legal report of a distinguished physician of Rochelle, at the time when the civil tribunal had been required to pronounce the judgment which should modify his civic *status* and replace him in his true sex. This report being very accurate and well drawn, I give it entire, and should have little to add thereto, as far as concerns the external genital organs, had not some modifications supervened during the time which separated the two examinations. The following is the report as it appears in the *Annales de Médecine légale* :*

"I, the subscriber, Doctor in Medicine, residing at la Rochelle, Department of la Chareute Inférieure, state the following to whom it may concern :

"A child, born of the married couple B., at St. Jean-d'Angely, the 8th of November, 1838, was declared at the civic registration to be a girl, and although registered under the names Adelaide-Herculine, her parents acquired the habit of calling her Alexina, a name which she has continued to bear up to this moment. Placed in female schools, and later, in the normal school of the Department of la Chareute-Inférieure, Alexina secured, two years ago, a teacher's certificate, and performed the functions in a boarding-school.

"Complaining of pains which she experienced in the left groin, it was decided to subject her to an examination by a physician who could not repress an expression of surprise at the sight of the genital organs. He communicated the result of his observations to the mistress of the school, who endeavored to tranquilize Alexina, by telling her that what had been observed belonged to her organization, and that there was nothing about which she

* *Annales d'hygiene et de Médecine légale.* 1860. 2nd séri, t. xiv., p. 206 — Question d'identité; vice de conformation des organs genitaux externes; — hypospadias; erreur sur le sexe, par le docteur Chesnet (de la Rochelle).

should be alarmed. Alexina, preoccupied, however, with a sort of mystery, of which she perceived herself to be the object, and with some expressions which escaped from the physician during his visit, commenced to scrutinize herself more attentively than she had hitherto done. In daily intercourse with young girls of fifteen to sixteen years, she experienced emotions which she controlled with difficulty. More than once during the night her dreams were accompanied by indefinable sensations. She felt herself moistened, and discovered, in the morning, grayish stains upon her linen.

"Surprised, as well as alarmed, Alexina confided this novel state of her mind to an ecclesiastic, who, no less astonished, doubtless, induced her to take advantage of a journey which she was about to make to la Rochelle, where her mother resided, to consult the bishop.

"She, in fact, presented herself to the bishop, and at the termination of this visit, I was instructed to examine Alexina with care, and to give my opinion upon her true sex. From this examination result the following facts:

"Alexina, who is in her twenty-second year, is of dark complexion; her height is $1.59^{m}$ (5 ft. $2\frac{1}{2}$ in.) The features of her face present nothing very characteristic, and remain undefined between those of a man and those of a woman. The voice is habitually feminine; but at times, in conversation or in coughing, there are mingled with it tones grave and masculine. A slight down covers the upper lip; some hairs of beard are perceptible upon the cheeks, especially upon the left. The chest is that of a man; it is flat, and without any appearance of mammæ. The menses have never appeared, to the great distress of her mother and of the physician whom she has consulted, and who has found his entire skill powerless to superinduce the appearance of this periodical flow. The upper limbs have not the rounded form which characterizes those of well-formed women; they are very brown, and slightly hairy. The pelvis and the hips are those of a man.

"The sub-pubic region is furnished with the most abundant black hair. If the legs are separated, a longitudinal slit is perceived, extending from the sub-pubic eminence to the border of the anus. At the upper part there is a peniform body, four to five centimetres in length from its point of insertion to its free extremity, which has the form of a glans covered with a prepuce, slightly flattened below, and imperforate. This little member, as far removed by its dimensions from the clitoris as from the penis in its normal state, can, according to the statement of Alexina, swell, harden and elongate. However, the erection, properly speaking, can be only limited, this imperfect penis being bound down inferiorly by a sort of bridle, which leaves only the gland free.

"The labia majora, apparent as may be observed upon each side of the slit, are very prominent, especially the right, and are covered with hair. They are, in reality, only the two halves of a scrotum remaining divided. They are, in fact, manifestly felt to be, upon handling them, an ovoidal body, suspended to the cord of spermatic vessels. The body, a little less developed than in the adult man, seemed to us to be nothing else than the testicle. Upon the right it had altogether descended. On the left it had remained higher; but it is movable, and descends more or less when it is pressed. These two globular bodies are very sensitive to pressure when it is somewhat strong. It was apparently the tedious passage of the testicle through the inguinal ring which occasioned the severe pains of which Alexina complained, and rendered necessary the visit of the physician, who, learning that Alexina had never menstruated, exclaimed: "I believe it; and she never will!"

"At the distance of a centimetre below the penis, is an urethra entirely feminine. I introduced into it a sound, and permitted the escape of a small quantity of urine. The sound being withdrawn, I induced Alexina to urinate in my presence, which was accomplished by a vigorous jet, directed horizontally to the outlet of the canal. It is very probable that the semen might also be ejected to a distance.

"Lower than the urethra, and at about two centimetres lower than the anus, is found the orifice of a very narrow canal, into which I could have succeeded in introducing the extremity of my little finger had not Alexina withdrawn, and seemed unable to bear the pain. I then introduced a female sound, and ascertained that this canal was five centimetres in depth, and terminated in a *cul-de-sac*. My index finger, introduced into the anus, detected the point of the sound through the walls which might be termed recto-vaginal. This canal is, then, a sort of vaginal outlet, at the bottom of which no vestige of a uterine neck can be detected. My finger, carried very high into the rectum, could not, through the parietes of the rectum, detect the uterus. The thighs and the legs, on their posterior portion, are covered with an abundance of black hair, as in the most hirsute man. From the above facts what shall we conclude? Is Alexina a woman? She has a vulva, labia majora, a female urethra, independent of a sort of imperforate penis. May not this be a clitoris monstrously developed? There is a vagina, very short indeed, very narrow, but, in fact, what is it if it is not a vagina? These are all feminine attributes. True, but Alexina has never menstruated; all the exterior of the body is that of a man. My examinations have not enabled me to detect a womb. Her tastes, her inclinations, attract her towards women. At night, voluptuous sensations are followed by spermatic emissions, the stained and thickened. To sum up, finally, ovoidal bodies, a cord of spermatic vessels are manifest to the touch in a divided scrotum. "These are the true evidences of sex. We may, therefore, conclude by saying: Alexina is a man; hermaphrodite, without doubt, but with evident prodominance of the masculine sex. Its history is, in its essential parts, the nearly complete reproduction of a case narrated by M. Marc, in the *Dictionaire des Sciences Médicales*, in the article, 'Hermaphrodite,' and cited, likewise, by Orfila, in the first volume of his *Médicine legale.* Marie Marguerité, of whom these authors speak, has solicited and obtained, from the tribunal of Dreux, the rectification of her sex upon the civic registers."

At the time when I proceeded to the examination of the cadaver, the report which I have just read had been made eight years, and the individual who was its subject, was in his thirtieth year.

The following is the condition which this unfortunate being presented in his miserable retreat, similar to which too many exist still in Paris, to be removed perhaps by the incessant progress of hygiene. A miserable pallet, a little table, and a chair, formed all the furniture of the place, in which four persons could scarcely stand. A little earthen furnace, in which only ashes remained, was found in a corner by the side of a rag containing wood charcoal. Upon the bed, the body was placed upon its back, partially dressed; the face was cyanosed, and an efflux of black and frothy blood occurred through the mouth. The height was the same as that noted in the report of M. Chesnet; the hair was black, quite abundant, and fine; the beard was equally black, but was not very abundant upon the lateral portions of the face. It was much thicker upon the chin, and upon the upper lip. The neck was small, and quite long, and the larynx was but little prominent forward. The voice, according to information gathered from those who had seen him, was not strongly vibratile. The chest had the ordinary dimensions and conformation of that of a man of this height, and no hair could be found upon it except around the nipples, which were black, and but slightly prominent. As to the breast, there was no more than is usual in a man of the same degree of fatness.

The lower and upper limbs were covered with very fine black hair, and the muscular protuberances were more decided than they are in women. The knees were not inclined towards each other; the feet and hands were small; the pelvis was not more developed than it should be in man.

STATE OF THE GENITAL ORGANS.

Upon the mons-veneris, which was prominent, there was an abundance of black hair, long and curled, which covered, also, the perineum, and the parts which simulated the labia majora, completely surrounding the anus — an arrangement which is altogether absent in women. In its normal position is seen a penis, regularly inserted, of 5 (1.95 in.) centimetres in length, and of 2½ (.97 in.) centimetres in diameter, in its flaccid state. This organ was terminated by an imperforate glans, flattened laterally, and completely uncovered by prepuce, which formed a crown at its root. This penis, which did not exceed in volume the clitoris of certain women, was slightly recurved beneath, being retained in this position by the inferior portion of the prepuce, which is finally intermingled with and lost in the folds of the skin which form the labia majora and minora.

A little below the penis, and in the same position which it occupies in the female, was found an urethra analogous to this latter. It is easy to introduce through this, a sound, and thus to reach the bladder, which we emptied in this manner. Lower than the urethra was seen the orifice of the vagina, and at the moment when we made the examination, there was a slight efflux of blood from the vulva. Doctor Regnier, who also recognized it, believed that it was occasioned by the repeated introduction of the finger.

This was indeed the only explanation admissible for this phenomena, the subject of the inquest never having had, as has been already explained, periodical effusions of blood from the vulva; and the examination of the internal organs readily furnishes the explanation of this.

The index finger could be readily introduced throughout the whole length of the vagina; but nothing was felt at the extremity of the finger which recalled the uterine neck. There was recognized, on the contrary, a *cul-de-sac.*

The length of this vagina was 6½ centimetres (2.5 in.) Upon the lateral portions, and throughout its whole length, the touch detected two small hard cords, placed below the mucous surface, and which were, as we shall describe hereafter, the ejaculatory ducts which finally open, one upon each side of the vulvar orifice. The vaginal mucous membrane was smooth and much injected, and was perceived to be covered, over its whole extent, with tesselated epithelium, such as that which invests the female vagina. The existence of little follicles was determined in the thickness of this mucous membrane. Near the vulvar orifice were found some circular folds of mucous membrane, but they do not suggest, by their arrangement, the existence of a hymen. In the space comprised between the folds of the prepuce, which hold the glans downward, and the vulvar orifice, were found quite a large number of small orifices, of excretory canals of glands situated beneath, and by compressing slightly the skin of this region, a gelatinous, colorless matter could be expressed from these little holes, which was nothing else than inspissated mucus.

The anus was situated 3½ centimetres from the vulva, and presented nothing abnormal. Upon each side of the erectile organ (penis or clitoris), and forming a veritable groove, in which this lay, were two voluminous folds of skin, which were the two lobes of a divided scrotum. The right lobe, much more voluminous than the left, contained, evidently, a testicle of normal size, and of which it was easy, through the skin, to trace the cord up to the ring. The left testicle had not completely descended, a large portion being still engaged in the ring.

### EXAMINATION OF INTERNAL ORGANS.

Upon opening the body, it was seen that the epididymis alone of the left testicle had escaped from the ring. It was smaller than the right. The vasa deferentia approximated behind and below the bladder. They had normal relations with the vesiculæ seminales, whence arose the two ejaculatory ducts, which made their exit and penetrated under the vaginal mucous membrane of each side as far as the vulvar orifice. The seminal vesicles, of which the right is a little more voluminous than the left, were distended with semen, which was of normal color and consistence. Microscopic examination of this liquid exhibited no spermatozoa, either in that taken from the vesiculæ, or in that from the testicles. Moreover, in that testicle which had passed the ring, and in the corresponding vesicle, some voluminous round bodies were seen, which resembled the mother-cells of the spermatozoa, or male ovules of M. Robin. It was easy to unroll the tubuli of both testicles, and the microscope showed nothing abnormal in that of the right side, but in that of the left, which was partially in the abdomen, the tubuli were fatty, and the parenchyma of the testicle had a yellowish tint that the other had not.

A little canula being placed in each of the vesiculæ seminales, I introduced an injection of milk in order to ascertain positively the direction of the ejaculatory ducts. This milk escaped, by jets, at the orifice of the vulva, upon each side, as I have already indicated. The bladder, in its normal position, was large. Distended by an injection of water, it extended above the pubis. Nothing suggested by its form the presence of ovaries or uterus. There was recognized, however, much above the *cul-de-sac* which forms the vagina, a thick, fibrous patch, upon which were displayed the vesiculæ seminales, which extend very high behind the bladder, and retains the vagina in a fixed position, on each side, suggesting, up to a certain point, the form of the broad ligaments; but the most attentive dissection does not admit the determination of any assimilation to an uterus or ovaries. It was, moreover, impossible to detect any orifice at the bottom of the vagina. It terminated in a complete *cul-de-sac*. The relations of the peritoneum with the bladder were normal, and it passed much above the vaginal *cul-de-sac*, and did not nearly touch its fundus.

The presence of two valvo-vaginal glands was readily determined by dissection, having their normal position and volume, as well as their little excretory conduits, opening a little below the ejaculatory seminal ducts. Upon pressing these glands, quite a large quantity of viscous liquid escaped.

Upon the urethra, and in the neighborhood of the neck of the bladder, there was likewise found a little gland, which was certainly a prostate, but slightly developed.

### DISCUSSION OF THE PRECEDING FACTS.

While it might appear extraordinary that an error about the sex of an individual could have been prolonged during so long a period, science nevertheless possesses quite a number of examples, of which some exhibit the closest analogy to the one under discussion. It is true, indeed, that the majority of these cases had not been the objects of attentive examination on the part of the physicians, and the physiological demonstration of the two sex was due most frequently to a fortuitous circumstance. One case may be remembered, " cited apropos of a memoir of Geoffroy Saint-Hilaire, of an hermaphrodite monk, considered as a man, and who, in spite of his vows of chastity, disclosed, in parturition, that his sex was not the same as that of his companions of the cloister." (L. Le Fort. *Vices de conformation des organes genitaux.*)

Schweikhard reports likewise the history of an individual registered as a girl, and considered as such up to the moment when he asked in marriage a young girl already *enciente* by him. In this individual the gland is imperforate, and the urethra opened below it; the urine, in escaping, following the horizontal direction of the penis. The author does not mention whether he was enabled to determine the place of emission of the semen.

Louis Caspar, in a treatise analysed by Martini, relates that " upon the complaint of a pregnant woman, who accused a midwife of having done her violence, and of having had sexual intercourse with her, the midwife was examined. It was determined that the clitoris, although more developed than usual, had not sufficient size to effect coition; that the vagina was so narrow that the extremity of the little finger only could be inserted into it, and there existed upon one side a little tumor, which might be supposed to be a testicle."

It would be easy to multiply examples of this sort; and it would be even profitable to science that all the documents which it possesses upon this question should be collected into compendium, which would become a precious guide to physicians who might be called upon to give opinions or to pronounce judgment upon those who should be involved in this anomalous condition. It would be readily apparent from such a work, that if it is sometimes difficult and even impossible at birth to recognize the true sex of individuals, the same difficulty would not exist at an advanced age, and especially near the age of puberty. There would be manifested at this period, in persons who have been the victims of an error, predilections and habits which are those of their true sex, the observation of which would aid considerably in designating their social position, if the condition of the genital organs and of their different functions should not prove sufficient to reach the same end. From such a reunion of observations, this fact would finally be clearly deduced, should its demonstration be still necessary, *that hermaphrodism does not exist in man and in the higher animals.*

Surgery is often all-powerful to rectify certain vices of conformation designated under the name hermaphrodism, and several cases of remarkable success are found reported in the thesis of M. Leon Le Fort, amongst others, that of Louise D——, borrowed from reports of M. Haguier, and for whom this surgeon made an artificial vagina with complete success. The case of Marie Madeleine Le Fort will be remembered, upon which Beclard was commissioned to report in 1815, and who died in 1864, at l'Hotel Dieu. Notwithstanding the very accurate report of Beclard, who decided that she was a woman, she was nevertheless considered, during forty years, by the majority of the physicians and surgeons of the Hospitals, who had opportunities for observing her in the different clinics at which she was presented, as belonging to the male sex. The autopsy, made by M. Dacorogna, interne of the ward in which Marie Madeleine Lefort died, not only demonstrated that Beclard was right, and that she possessed all the attributes appropriate to the sex to which he had assigned, and though she differed from other women only by a clitoris more voluminous than it should be, and an imperforation of the vagina, which was closed by a rather thick membrane, and the simple incision of this membrane would have sufficed to restore the subject definitely to her sex. Beclard had, moreover, proposed this operation at the time of making his examination.

For a long time many different causes have been adduced to explain this kind of anomaly. The aid of comparative anatomy, especially, has been frequently invoked; but since the brilliant labors of M. Coste, and of other modern embryogenists, it has been particularly of developmental anatomy, or embryogenesis, that the light necessary to resolve similar questions has been sought. It is indeed the study of embryogenesis which teaches us that the different stages of arrest sustained by embryos form the origin of different deformities or monstrosities which are only too frequently submitted to our observation, and which constitute, to a great extent, pathological anatomy, and the whole science of monstrosities or terratology. I shall then lay under contribution the science of embryogenesis, to explain the state of the external genital organs of the subject of this report. According to M. Coste, the external genital organs only commence to make their appearance from the fortieth or forty-first day, and then that the corresponding internal organs have already commenced their development several days before. There may be perceived, then, at this fœtal period, at the base of the caudal protuberance, in the little slit which is more and more hollowed out, and which should, at a later period, communicate with the bladder, the vagina and the rectum, — there may be perceived, I repeat, at the upper extremity of this little slit or groove, two little roundish bodies, which should, in man, constitute the origin to the corpora cavemosa penis, and in woman to the clitoris and the labia minora.

These two little eminences are united by their superior margins, and form, between their inferior margins, which remain free, a little gutter which should remain permanently in woman, but in man is transformed into a complete canal, and constitutes the urethra. The absence of this union, in man, of the free margins of this slit or gutter, establishes the vice of conformation which is designated by the name hypospadias, which is the condition of the subject now under examination. Below these little eminences just referred to, two other are soon developed, which should form the scrotum of man or the labia majora of women. It is, therefore, the non-union of the two lobes of the scrotum which constitutes what I have designated under the name of labia majora in the subject under examination. The analogy which may be established between the different glands which are found in the vagina of woman and those of the male urethra authorizes as perfectly, to affirm that the valvo-vaginal glands of our subject were no other than those of Cowper, or the bulbo-urethral glands; those in the vagina which terminated in a cul-de-sac, were the urethral glands of man. This vaginal cul-de-sac itself was nothing else than the urethra, which should have remained in its normal state.

Professor Courty, who has extensively investigated the organic analogies existing in different mechanisms, justifies likewise, in a very clear and plausible manner, those which he establishes between the membranous portion of the urethra in man and the vagina in woman. "The vagina, indeed, is developed in the blastema, intermediate to the bladder and the rectum, immediately below the middle perineal aponeurosis, by the formation in the vesico-rectal interspace, of a canal which extends until it meets at one extremity the vulvar opening, and at the other the uterine neck. It is at the same identical point, and in the same manner, that the membranous portion of the urethra of man is formed in front of the urethral crest (ridge formed by the approximation of the two seminal ducts), and behind the groove or gutter of the penis, which is soon converted into a canal by means of an inferior joint extending to the bulb, inclusive.

"From this analogy, confirmed elsewhere by all sorts of proofs which cannot be reproduced here, there is evolved a consequence which permit an apparent paradox at first sight; that is to say, that in man there is properly speaking no urethral canal, whilst there is really one in woman. In man the canal through which the urine escapes from the bladder is nothing else than the analogue of the vulvo-vaginal canal of woman, developed differently and accommodated to other uses. In man the urinary passages, strictly speaking, terminate at the neck of the bladder. The canal which continues thence belongs, by its origin and termination, to the genital apparatus. It is, in truth and pre-eminently, the ejeculator of semen. *It only lends itself* to the excretion of urine, this liquid traversing it from one end to the other, passing successively through its prostatic portion (uterine neck, its membranous (vagina), and its bulbo-spongiose (vestibule); additional proof of the differences of structure or of destination which nature can impress upon organs fundamentally identical." *

The position of the ejaculatory ducts in the subject of this report confirms the theory of M. Courty; it is apparent, indeed, that in the normal development of this urethra transformed into a vagina, the external orifice of these little canals would correspond to the situation of the verumontanum.

Among the medico-legal questions which might be raised by a case similar to that of Alexina, is that in which an expert might be called upon to pronounce upon the aptitude for marriage and reproduction. It would be assuredly very embarrassing to decide such a question; but I do not believe that there would have been sufficient authority, after a careful examination of the genital organs, to decide negatively in the one case or the other. Procreation being the natural end of marriage, Alexina was the bearer of organs characteristic of his sex, whose functions were performed. The disposition of the ejaculatory ducts was contrary to that which permitted the passage of the semen directly to the bottom of the vagina; but it is well understood to-day that fecundation may result even when the seminal fluid impregnates only the entrance to the vagina. Science possesses numerous examples of subjects affected by hypospadias, whose external urethral orifice was more or less approximated to the scrotum, who nevertheless became the fathers of several children; and in these cases the authenticity of the paternity was demonstrated by the hereditary transmission to their children of the vices of conformation of which they themselves were the subjects.

* A. Courty, *Maladie de l'uterus et de ses annexis.* Paris. 1867, in -8, p. 37.

The seminal fluid taken from the vesicle corresponding to the descended testicle, in the subject of our notice, contained no spermatozoa; for a much stronger reason, the semen taken from the vesicle of the testicle which remained engaged in the ring should likewise have been destitute of it,* and this appears to be the rule for testicles which do not accomplish their complete migration; but this state of affairs could be only temporary in the case of Alexina, for the testicle which had completely descended, and at some future time the existence of spermatozoa in the seminal liquid might have been established. It is well known that, in men who have every appearance of health, there is sometimes a temporary absence of spermatozoa, under some peculiar influence, and that they may reappear subsequently. This might very well occur in the subject of our notice. Contrary to the cases of Follin, the numerous and interesting cases of monorchidia reported by E. Godard, demonstrate in a constant manner the presence of spermatozoa in the seminal fluid of individuals who had but one testicle in the scrotum.

* Follin has likewise reported his observations of individuals who had but one testicle in the scrotum, and in whom no spermatozoa could be found either in one side or the other. (See also the researches of Godard, *Sur la monorchidie et la cryptorchidie*, 1 vol., in -8, 1860; et, *Comptes Rendus et memoires de la Société de Biologie*, 1859, with plates.)

